# Exploratory Whole-Genome Profiling of Greek Men with Oligozoospermia: Identification of Candidate Variants, Genes, and Biological Pathways

**DOI:** 10.3390/genes17101187

**Published:** 2026-09-25

**Authors:** Maria-Anna Kyrgiafini, Georgia Vetsika, Georgia Patsali, Aris Kaltsas, Georgios Stamatellos, Zissis Mamuris

**Affiliations:** 1 Laboratory of Genetics, Comparative and Evolutionary Biology, Department of Biochemistry and Biotechnology, University of Thessaly, Viopolis, Mezourlo, 41500 Larissa, Greece; gvetsik@gmail.com (G.V.); gpatsale@gmail.com (G.P.); stamatellosgeo@gmail.com (G.S.); 2 Third Department of Urology, Attikon University Hospital, School of Medicine, National and Kapodistrian University of Athens, 12462 Athens, Greece; ares-kaltsas@hotmail.com

**Keywords:** male infertility, whole-genome sequencing, sperm, cilia, axoneme

## Abstract

Background/Objectives: Oligozoospermia is a common cause of male infertility, yet its genetic basis remains incompletely understood, particularly in underrepresented populations. This exploratory pooled-WGS study aimed to identify and prioritize candidate coding variants, genes, and biological pathways of potential relevance to oligozoospermia in Greek men. Methods: Whole-genome sequencing was performed on genomic DNA from Greek normozoospermic and oligozoospermic men. Variants detected in the oligozoospermic group but not in the normozoospermic group within the present dataset were prioritized according to predicted functional impact, population allele frequency, evolutionary conservation, and multiple in silico pathogenicity prediction tools. Gene Ontology (GO), Kyoto Encyclopedia of Genes and Genomes (KEGG), and STRING protein–protein interaction analyses were subsequently performed to investigate the biological significance of the prioritized genes. Results: A total of 717,374 variants were identified exclusively in oligozoospermic men. Following prioritization, 171 coding variants (54 high-impact and 117 moderate-impact) mapped to candidate genes of potential biological relevance to male infertility, including 22 variants not previously observed in the databases examined. Functional enrichment analyses consistently highlighted biological processes related to cytoskeletal organization, microtubule dynamics, axonemal assembly, and ciliary function. Several genes previously implicated in male infertility, including *DNAH5, DNAH11*, and *DNAH12*, were prioritized alongside promising candidate genes such as *COL6A6* and *LAPTM4B*. Conclusions: This study provides one of the first whole-genome investigations of oligozoospermia in a Greek population and expands the limited genomic data available for Balkan populations. However, given the exploratory pooled-WGS design and limited cohort size, these findings should be considered hypothesis-generating and require validation in larger, individually sequenced cohorts.

## 1. Introduction

Infertility, as defined by the World Health Organization (WHO), is the inability to achieve pregnancy after 12 months or more of regular, unprotected sexual intercourse [1]. Beyond its profound psychological and social impact on affected couples, infertility also imposes a substantial economic burden on both patients and healthcare systems [2,3,4]. According to the Global Burden of Disease study, infertility affected an estimated 55.0 million men and 110.1 million women worldwide in 2021, with prevalence continuing to increase globally and projected to rise further over the coming decades [5]. Male factors contribute to nearly half of all infertility cases, either as the sole cause or in combination with female factors, underscoring the importance of understanding the biological mechanisms underlying male reproductive dysfunction [6].

Based on semen analysis, male infertility can be classified into distinct clinical phenotypes according to the affected sperm parameter, including asthenozoospermia (reduced sperm motility), teratozoospermia (abnormal sperm morphology), and oligozoospermia (reduced sperm concentration) [7]. Oligozoospermia is defined as a sperm concentration below the lower reference limit established by the WHO. These reference limits have been periodically revised as evidence from fertile populations has accumulated, reflecting the evolving understanding of semen quality and male reproductive potential [8,9]. In the latest WHO Laboratory Manual for the Examination and Processing of Human Semen (6th edition), the lower reference limit for sperm concentration is set at 16 million spermatozoa/mL, representing the fifth percentile of fertile men [8].

Although advances in semen analysis have improved the clinical classification of male infertility, the genetic architecture of oligozoospermia remains far from fully understood. Male infertility is recognized as a multifactorial disorder resulting from the interplay of genetic, environmental, endocrine, and lifestyle factors [7]. Genetic abnormalities are estimated to account for at least 15% of male infertility cases, a proportion likely underestimated given that many causative variants remain unidentified [10,11]. Spermatogenesis is a highly coordinated biological process involving the expression of more than 2000 genes that regulate mitotic proliferation, meiosis, and post-meiotic sperm differentiation, highlighting the remarkable genetic complexity underlying normal sperm production [11]. Consequently, identifying the specific genetic defects responsible for impaired spermatogenesis remains a considerable challenge. While routine genetic testing—including karyotype analysis, Y chromosome microdeletion screening, and *CFTR* testing in selected cases—has substantially improved the diagnosis of male infertility, a large proportion of men with oligozoospermia still receive an idiopathic diagnosis following standard clinical evaluation [7]. Furthermore, accumulating evidence suggests that semen characteristics and reproductive outcomes may vary among different ethnic populations; however, the contribution of population-specific genetic variation to male infertility remains largely unexplored [12,13]. These observations underscore the need for comprehensive genomic studies in diverse populations to identify novel genetic variants associated with oligozoospermia.

Recent advances in next-generation sequencing (NGS), particularly whole-genome sequencing (WGS), have substantially expanded our ability to investigate the genetic basis of complex diseases [14]. Unlike SNP arrays and genome-wide association studies (GWAS), WGS enables the comprehensive detection of both common and rare genetic variants across the entire genome, providing an unbiased approach for identifying variants that may contribute to disease susceptibility [15]. Beyond conventional semen analysis, molecular and omics-based approaches can provide complementary information on biological alterations relevant to male reproductive function. For example, Ferrero et al. (2024) [16] demonstrated that small RNA sequencing and molecular profiling of male germ cells identified differences in sperm small non-coding RNAs and other molecular features associated with environmental exposure, even when conventional sperm concentration and motility were comparable between groups [16]. Such findings highlight the complementary value of molecular profiling and support the application of genome-wide approaches, including WGS, to further investigate the complex biological basis of male infertility. Given the considerable genetic diversity among human populations and the limited genomic data currently available from Balkan populations, comprehensive genomic studies are essential to identify both shared and population-specific genetic determinants underlying distinct male infertility phenotypes, particularly oligozoospermia.

Thus, the aim of this study was to characterize the whole-genome variant profile of Greek men with oligozoospermia and identify candidate coding variants, genes, and biological pathways of potential relevance to oligozoospermia through whole-genome sequencing and functional enrichment analyses. By generating genomic data from an underrepresented European population, this study provides preliminary insights into the genetic architecture of oligozoospermia and establishes a valuable resource for future genetic and functional studies of male infertility.

## 2. Materials and Methods

### 2.1. Study Population and Sample Collection

Study participants were recruited in Greece and provided peripheral blood and semen samples for clinical and genetic analyses. The study protocol was approved by the Ethics Committee of the University of Thessaly (Volos, Greece), and written informed consent was obtained from all participants prior to enrollment.

All participants underwent a comprehensive andrological evaluation at an accredited andrology laboratory operating under standardized quality-controlled procedures. Semen samples were obtained by masturbation following 2–5 days of sexual abstinence and were analyzed according to the WHO Laboratory Manual for the Examination and Processing of Human Semen (6th edition, 2021) (https://www.who.int/publications/i/item/9789240030787, accessed on 3 August 2026). Semen volume, sperm concentration, total and progressive motility, and sperm morphology were assessed using WHO reference criteria, and the results were used to classify participants according to their semen phenotype. To minimize the impact of the natural intra-individual variability of semen parameters, abnormal findings were confirmed by repeated semen analyses performed 3–6 months after the initial evaluation. Oligozoospermia was defined as a sperm concentration below 16 × 10^6^ spermatozoa/mL, corresponding to the fifth centile reported in the sixth edition of the WHO Laboratory Manual for the Examination and Processing of Human Semen (2021). All participants classified as oligozoospermic exhibited progressive motility and normal morphology values above the corresponding WHO fifth centiles, allowing the selection of an isolated oligozoospermic phenotype rather than a mixed semen phenotype. Normozoospermic participants had semen parameters at or above the corresponding WHO lower fifth centiles, including sperm concentration ≥ 16 × 10^6^/mL, total sperm number ≥ 39 × 10^6^ per ejaculate, progressive motility ≥ 30%, total motility ≥ 42%, and normal morphology ≥ 4%.

To ensure that only cases of idiopathic male infertility were included, strict exclusion criteria were applied. Detailed medical and reproductive histories, including medication use, were obtained for all participants. Individuals with known endocrine disorders or receiving treatments known to interfere with spermatogenesis were excluded. Individuals with varicocele, reproductive tract infections, testicular trauma or other testicular pathologies, cryptorchidism, orchitis, epididymitis, endocrine disorders (including diabetes mellitus, thyroid disease, and hypogonadism), autoimmune diseases, metabolic syndrome, active or previous malignancies, or other medical conditions known to impair spermatogenesis were also excluded. Systematic hormonal measurements were not performed as part of the present study; therefore, hormonal status was assessed only through the identification and exclusion of known endocrine disorders based on the participants’ clinical and medical history. In addition, all participants underwent genetic evaluation to exclude known chromosomal abnormalities and Y chromosome microdeletions. No separate comprehensive screening for known infertility-associated sequence variants was performed prior to WGS. Individuals with azoospermia were not included in the study. Among the oligozoospermic participants included in the final cohort, sperm concentrations ranged from 6.8 to 12.3 × 10^6^ spermatozoa/mL. The normozoospermic controls had normal semen parameters according to the WHO reference criteria stated above and a documented history of at least one pregnancy.

A total of 15 unrelated Greek men were included in the study, comprising 10 normozoospermic controls and 5 men diagnosed with oligozoospermia. Only individuals of Greek ethnicity were included in the study. This criterion was adopted to reduce population heterogeneity and to address the limited availability of genomic data related to male infertility in Greek and Balkan populations. In parallel, demographic and lifestyle information, including age, body mass index (BMI), smoking habits, alcohol consumption, place of birth, geographic origin, and medication use, were collected through questionnaires completed during enrollment. A summary of participant characteristics is presented in Table 1. Individual demographic, lifestyle, and semen-analysis characteristics of all study participants, including age, BMI, smoking status, alcohol consumption, abstinence period, semen volume, sperm concentration, total sperm count, progressive and total motility, normal morphology, vitality, and semen pH, are provided in Supplementary Table S1.

Given the potential influence of demographic and lifestyle factors on semen characteristics, age, BMI, smoking status, and alcohol consumption were considered as potential confounding factors and compared between the two study groups. The normozoospermic and oligozoospermic groups had mean ages of 36.4 ± 7.2 and 39.0 ± 2.8 years, respectively, and mean BMI values of 27.0 ± 6.1 and 31.1 ± 4.4 kg/m^2^, respectively. Statistical comparisons yielded *p*-values of 0.34 for age and 0.16 for BMI, while comparisons of smoking status and alcohol consumption yielded *p*-values of 1.00 and 0.33, respectively (Table 1).

### 2.2. DNA Extraction and Whole-Genome Sequencing

Genomic DNA was extracted from peripheral blood samples using the PureLink Genomic DNA Mini Kit (Invitrogen, Waltham, MA, USA; Cat. No. K182002) according to the manufacturer’s protocol. DNA quality was subsequently assessed by agarose gel electrophoresis, while DNA concentration was determined using a Qubit 2.0 Fluorometer and the Qubit dsDNA BR Assay Kit (Invitrogen, Waltham, MA, USA; Cat. No. Q32850).

To reduce sequencing costs while maintaining adequate coverage, samples were analyzed using a pooled whole-genome sequencing strategy. Specifically, DNA from ten normozoospermic individuals was divided into two pools of five individuals each, whereas DNA from five oligozoospermic individuals was combined into a single pool. Accordingly, within each pool, DNA samples were mixed in equimolar proportions to a final concentration of 100 ng/µL and a final quantity of 2 mg.

Pooled DNA libraries were prepared and sequenced by Novogene (Cambridge, UK). Specifically, paired-end libraries (2 × 100 bp) were generated and sequenced on the Illumina HiSeq 3000 platform (Illumina, Inc., San Diego, CA, USA) to an average depth of approximately 30×.

### 2.3. Bioinformatics Analysis and Variant Identification

Raw sequencing reads were initially assessed using FastQC v0.12.1 (available online at: http://www.bioinformatics.babraham.ac.uk/projects/fastqc/, accessed on 3 August 2026). Adapter sequences and low-quality bases (PHRED score < 30) were removed using Trimmomatic v0.39 [17]. Subsequently, filtered reads were aligned to the human reference genome GRCh38/hg38 retrieved from the Ensembl database [18] using the Burrows–Wheeler Aligner (BWA) v0.7.17 [19]. PCR duplicates were identified and removed using Picard tools, and alignment files were converted to BAM format using SAMtools v1.21 [20]. Finally, BAM files corresponding to the two normozoospermic pools were merged into a single representative dataset using SAMtools [20].

Variant calling was performed using FreeBayes [21], generating variant call format (VCF) files for both study groups. Specifically, variant calling was performed using FreeBayes v1.3.1 [21] with parameters adapted for pooled sequencing. The oligozoospermic dataset comprised pooled DNA from five diploid individuals, whereas the two normozoospermic pools, each comprising five individuals, were merged prior to variant calling. Accordingly, variant calling was performed in pooled-discrete mode, with autosomal ploidy set to 10 for the oligozoospermic pool and to 20 for the merged normozoospermic dataset. To retain sensitivity for low-frequency alleles within the pooled datasets, a minimum alternate allele fraction of 0.05 and a minimum of three alternate-supporting reads were required. Reads and bases with mapping quality < 30 and base quality < 20, respectively, were excluded from variant evaluation. Following variant calling, variants with sequencing depth < 20× or variant quality < 30 were excluded. For between-group comparisons, a variant detected in the oligozoospermic dataset was considered not detected in the normozoospermic dataset only when the corresponding genomic position in the merged normozoospermic dataset had sufficient sequencing coverage but did not meet the predefined alternate-read and allele-fraction criteria for variant detection. Positions with insufficient coverage were not considered evidence of absence. Then, comparative analysis of the normozoospermic and oligozoospermic VCF files was conducted using BCFtools [20] to identify variants exclusively present in the oligozoospermic pool. It should be clarified that for the purposes of the present study, variants were considered “oligozoospermia-exclusive” when they were detected in the pooled oligozoospermic WGS dataset but not in the merged normozoospermic WGS dataset. Accordingly, the term “exclusive” refers to the comparative variant profiles of the study groups within the present dataset and does not indicate that a variant was present in every individual comprising the oligozoospermic pool or that it is associated with the phenotype. Subsequent analyses focused on variants detected in the oligozoospermic pool but not in the merged normozoospermic dataset, as these represented group-specific variants within the present cohort and constituted the initial set for downstream prioritization.

Functional annotation of oligozoospermia-specific variants was performed using the Ensembl Variant Effect Predictor (VEP) (available at https://www.ensembl.org/Tools/VEP, accessed on 3 August 2026) [22]. Additional information regarding variant frequency was obtained from the Single Nucleotide Polymorphism Database (dbSNP) [23], the 1000 Genomes Project [24], and the Genome Aggregation Database (gnomAD) [25]. Furthermore, to evaluate the potential functional consequences of identified variants, multiple in silico prediction tools were employed, including Sorting Intolerant From Tolerant (SIFT) [26], Polymorphism Phenotyping v2 (PolyPhen-2) [27], Combined Annotation Dependent Depletion (CADD) [28], and MutationAssessor [29].

It should be noted that although variant calling was performed using WGS data, the downstream functional prioritization applied in the present study was specifically focused on nuclear coding SNPs and small indels, particularly variants annotated as having HIGH predicted impact or as missense variants. Non-coding variants were not subjected to systematic downstream prioritization. Dedicated analyses of mitochondrial DNA variants, structural variants (SVs), copy-number variants (CNVs), and repeat expansions were also not performed and were outside the predefined analytical scope of the present study.

An overview of the bioinformatics workflow is presented in Figure 1.

### 2.4. Variant Prioritization and Functional Characterization

To identify candidate variants with the greatest potential biological relevance, a systematic multi-step prioritization strategy was implemented (Figure 2). The workflow was designed to progressively reduce the number of candidate variants while enriching for rare variants with predicted functional relevance.

(A) High-impact variants. Variant consequences were annotated using the Ensembl Variant Effect Predictor (VEP) [22]. In the present study, the term ‘high-impact’ refers specifically to variants classified by VEP as having a HIGH predicted impact on the transcript or encoded protein, including consequences such as start-loss, stop-gained, frameshift, and essential splice-site variants. The VEP impact classification represents a prediction of the potential molecular consequence of a variant and does not constitute evidence of clinical pathogenicity or causality. Consequently, variants classified by the Ensembl Variant Effect Predictor (VEP) [22] as high impact were prioritized because these alterations are more likely to disrupt gene function and have been widely associated with diseases [30,31]. To focus on rare variants with potential functional relevance, common variants (minor allele frequency, MAF > 5%) were excluded based on allele frequencies from the 1000 Genomes Project (Phase 3) [24] and the non-Finnish European population of the Genome Aggregation Database (gnomAD) (v4.1) [25]. Given the exclusively Greek composition of the study cohort, European reference populations were used as the closest available approximation to its genetic background. Rare variants may be particularly informative in studies of complex diseases by facilitating the prioritization of less common genetic variation with potential functional relevance [32,33]. Because the present study focused exclusively on a Greek cohort, allele frequency filtering was based on European reference populations, which provide the closest available approximation to the genetic background of the study population, too. Furthermore, only variants with a CADD score > 10 [28], indicating a higher predicted potential for functional impact, were retained.

(B) Moderate-impact variants. Missense variants classified as moderate impact by VEP [22] were evaluated separately. Although these variants do not necessarily abolish protein function, they may alter protein structure or activity and contribute to complex disease susceptibility [34]. Therefore, only variants consistently predicted to be deleterious by multiple computational tools were retained, requiring a SIFT score ≤ 0.05 [26], a PolyPhen-2 score ≥ 0.80 [27], and a medium or high functional impact according to MutationAssessor [29]. The same rarity (MAF ≤ 5%) and CADD (>10) [28] thresholds applied to high-impact variants were also used for moderate-impact variants.

Variants not identified in Ensembl (release 109) [18], the 1000 Genomes Project (Phase 3) [24], or gnomAD (v4.1) [25] were classified as ‘not previously observed in the databases examined’ and were retained irrespective of population-frequency criteria. This classification refers exclusively to their absence from the queried databases at the time of analysis and does not imply true novelty or pathogenicity.

Following variant prioritization, the biological relevance of the affected genes was investigated through functional annotation using GO [35,36], KEGG [37], and GeneCards [38]. Protein–protein interaction (PPI) networks were further explored using STRING [39] to identify functional relationships among genes harboring prioritized variants. Finally, genes harboring multiple prioritized variants were highlighted for further investigation, as the presence of multiple variants with predicted functional relevance within the same gene provided an additional criterion for candidate-gene prioritization.

## 3. Results

### 3.1. Variant Calling and Annotation of WGS Data

Following whole-genome sequencing and variant calling, a comparative analysis was performed to identify variants exclusively detected in either the normozoospermic or oligozoospermic group. The available pool-specific sequencing quality metrics showed comparable performance across the three sequencing pools (Supplementary Table S2). A total of 717,374 variants were detected in the pooled oligozoospermic dataset but not in the merged normozoospermic dataset, whereas 2,260,073 variants were detected in the merged normozoospermic dataset but not in the oligozoospermic dataset. These variants mapped to 26,451 and 34,650 genes, respectively, including both protein-coding genes and non-coding genomic regions, such as long non-coding RNAs (lncRNAs) and microRNAs (miRNAs).

Given the objective of the present study, subsequent analyses focused exclusively on the 717,374 variants identified in the oligozoospermic group but not in the normozoospermic group. These variants were considered group-specific within the present dataset rather than statistically associated with oligozoospermia and constituted the initial set for subsequent functional and population-frequency-based prioritization. This prioritization was performed to identify candidate variants of potential biological relevance for further investigation.

### 3.2. High-Impact Variant Identification

Among the 717,374 variants detected in the pooled oligozoospermic dataset but not in the merged normozoospermic dataset, classification according to the highest-impact VEP [22] consequence assigned to each variant identified 203 HIGH-, 2474 MODERATE-, 8041 LOW-, and 706,656 MODIFIER-impact variants. Downstream prioritization focused on the HIGH- and MODERATE-impact categories because of their greater predicted potential to affect protein-coding genes.

Among the 203 variants classified as HIGH impact, 201 were retained following exclusion of common variants (MAF > 5%) based on European population-frequency data from the 1000 Genomes Project [24] and gnomAD [25]. These included 52 nonsense (stop-gained/stop-lost), 56 frameshift, 84 splice-disrupting, and 9 start-loss variants (Table S3). Subsequent application of the CADD >10 criterion reduced this set to 54 prioritized HIGH-impact variants.

The 54 prioritized high-impact variants comprised 17 nonsense, 17 frameshift, 18 splice-disrupting, and 2 start-loss variants (Table S4). Among the prioritized variants, 12 were not previously observed in the databases examined, namely Ensembl (release 109) [18], the 1000 Genomes Project (Phase 3) [24], and gnomAD (v4.1) [25]. Collectively, these variants mapped to 54 unique genes, which were subsequently subjected to functional enrichment and pathway analyses.

### 3.3. Moderate-Impact Variant Identification

A total of 2474 variants were classified as MODERATE-impact missense variants according to VEP [22] and were subjected to sequential prioritization. Application of the SIFT (≤0.05) and PolyPhen-2 (≥0.80) criteria reduced this set to 781 variants. Further filtering based on a medium or high functional impact predicted by MutationAssessor reduced the set to 452 variants. Following population-frequency filtering (MAF ≤ 5%), 307 variants, mapping to 301 genes, were retained (Table S5). Subsequent application of the CADD > 10 criterion resulted in 117 prioritized MODERATE-impact missense variants (Table S6). Among these, 10 variants were not previously observed in the databases examined, namely Ensembl (release 109) [18], the 1000 Genomes Project (Phase 3) [24], and gnomAD (v4.1) [25]. Collectively, the prioritized moderate-impact variants mapped to 117 unique genes, which were subsequently included in downstream functional analyses.

### 3.4. Functional Enrichment and Protein–Protein Interaction Analysis

To investigate the biological significance of the prioritized genes, Gene Ontology (GO), Kyoto Encyclopedia of Genes and Genomes (KEGG), and STRING protein–protein interaction (PPI) analyses were performed.

GO Biological Process (BP) enrichment analysis revealed a significant overrepresentation of genes involved in microtubule-based processes, cilium organization, cilium assembly, and axonemal dynein complex assembly (Figure 3). Additional enriched biological processes included the regulation of potassium ion transmembrane transporter activity and maintenance of organelle location, suggesting that the prioritized genes participate in diverse cellular mechanisms involved in cytoskeletal organization and intracellular transport.

GO Cellular Component (CC) analysis further demonstrated significant enrichment of structures directly associated with motile cilia and sperm flagella (Figure 4). The most significantly enriched cellular components included the 9 + 0 motile cilium, outer dynein arm, axonemal dynein complex, dynein complex, axoneme, ciliary plasm, microtubule cytoskeleton, and cytoskeleton. Additional enrichment was observed for cell projection, plasma membrane-bounded cell projection, and apical plasma membrane components, further supporting the involvement of genes responsible for cytoskeletal organization and flagellar architecture.

Consistent with these findings, GO Molecular Function (MF) analysis identified significant enrichment for minus-end-directed microtubule motor activity, primarily driven by the dynein heavy-chain genes *DNAH5*, *DNAH11,* and *DNAH12* (Table 2). These genes encode axonemal motor proteins that play essential roles in ciliary motility and sperm flagellar movement.

KEGG pathway analysis identified a significant enrichment of the Cytoskeleton in muscle cells pathway (Table 3). Although originally annotated in muscle tissue, this pathway includes several structural proteins involved in cytoskeletal organization and cell architecture, further highlighting cytoskeletal organization as a biological theme within the prioritized gene set.

To further investigate the functional relationships among the prioritized genes, a STRING protein–protein interaction (PPI) network was constructed (Figure 5). The resulting network comprised 168 nodes and 75 edges, significantly exceeding the 47 interactions expected by chance (PPI enrichment *p* = 1.12 × 10^−4^), indicating that the identified genes are functionally interconnected rather than randomly associated. Functional enrichment of the interaction network highlighted proteins involved in cytoskeletal organization (FDR = 0.0017) and cilium-associated processes, including genes implicated in Kartagener syndrome, further highlighting cytoskeletal and cilium-associated processes within the prioritized gene set.

Collectively, GO, KEGG, and STRING analyses of the prioritized gene set highlighted convergent biological themes related to cytoskeletal organization, microtubule dynamics, axonemal assembly, and ciliary function. Because these complementary analyses were derived from substantially the same prioritized gene set, their convergence should be interpreted as a hypothesis-generating pathway-level signal rather than as independent validation of these biological processes in oligozoospermia.

### 3.5. Genes Harboring Multiple Prioritized Variants

As an additional prioritization step, genes harboring more than one prioritized variant were identified. Two genes, *LAPTM4B* and *COL6A6*, each contained two independently prioritized variants, including both high- and moderate-impact alterations (Table 4). Specifically, *LAPTM4B* harbored one frameshift and one missense variant, whereas *COL6A6* contained one splice-disrupting and one missense variant. The presence of multiple prioritized variants with predicted functional relevance within the same gene further supported their prioritization as candidate genes for further investigation. Based on the available literature, the prioritized genes were further distinguished according to the extent of previous evidence linking them to male infertility or spermatogenic phenotypes. Accordingly, genes with previously reported involvement in male infertility or related reproductive phenotypes were considered separately from less-characterized candidate genes for which direct evidence of involvement in male infertility remains limited.

## 4. Discussion

The present exploratory pooled-WGS study provides an initial characterization of group-specific genetic variation and candidate biological pathways of potential relevance to oligozoospermia in Greek men. The analytical framework was designed for candidate discovery and prioritization rather than formal case–control association testing. By integrating variant prioritization with functional enrichment and protein–protein interaction analyses, we identified multiple rare variants, including variants not previously observed in the databases examined, that converged on biological processes related to cytoskeletal organization, microtubule dynamics, axonemal assembly, and ciliary function. Importantly, several of the genes harboring prioritized variants have previously been implicated in male infertility or reproductive disorders, providing additional support for the biological relevance of the identified candidate variants. Furthermore, complementary GO, KEGG, and STRING analyses highlighted overlapping biological themes within the prioritized gene set. In addition, some genes emerged as particularly promising candidates based on the presence of multiple prioritized variants. Collectively, these findings provide an exploratory basis for further investigation of candidate genetic variation and biological pathways potentially relevant to oligozoospermia and expand the currently limited genomic data available for Balkan populations. They also provide a valuable foundation for future validation studies aimed at clarifying the molecular mechanisms underlying impaired spermatogenesis and male infertility.

### 4.1. Cytoskeletal Organization, Axonemal Assembly, and Ciliary Function in Oligozoospermia

One of the prominent findings of the present study was the convergence of complementary functional analyses on biological themes related to cytoskeletal organization, microtubule dynamics, axonemal assembly, and ciliary function. Gene Ontology enrichment consistently highlighted processes related to microtubule-based organization, cilium assembly, and axonemal dynein complex formation. Concurrently, Cellular Component analysis demonstrated significant enrichment of structures directly associated with the sperm flagellum, encompassing the axoneme, dynein complex, outer dynein arm, and microtubule cytoskeleton. Similarly, STRING protein–protein interaction analysis revealed a significantly interconnected network enriched for cytoskeletal proteins, further highlighting related functional relationships within the prioritized gene set. Together, these findings suggest that disruption of the cytoskeletal machinery constitutes a central molecular feature of the oligozoospermic genomic profile identified in the present study.

The sperm flagellum is a highly specialized microtubule-based organelle whose formation depends on the coordinated assembly of the axoneme, molecular motor proteins, and numerous structural and regulatory components during spermatogenesis [40]. Beyond providing motility, proper cytoskeletal organization is essential throughout germ cell development, contributing to meiotic chromosome segregation, spermatid differentiation, acrosome biogenesis, manchette formation, and flagellar elongation [41,42]. Consequently, defects affecting cytoskeletal architecture or ciliary assembly can impair multiple stages of sperm development, ultimately leading to reduced sperm production and compromised semen quality.

Genomic studies have increasingly demonstrated that pathogenic variants in genes implicated in microtubule organization and ciliary biology are a significant genetic cause of male infertility, including oligozoospermia, asthenozoospermia, and multiple morphological abnormalities of the sperm flagella (MMAF). The observations from this study therefore align with existing research indicating that disruptions in cytoskeletal, ciliary, and axonemal genes contribute to a broad spectrum of male infertility phenotypes. Specifically, whole-exome sequencing of men with severe flagellar abnormalities has identified pathogenic variants in genes such as *CFAP43*, *CFAP44*, *CFAP69*, *DNAH1*, and *DNAH8*, with subsequent ultrastructural analyses revealing abnormalities of the axonemal microtubules. While these patients were predominantly characterized by severe asthenozoospermia and multiple morphological abnormalities of the sperm flagella, the frequent occurrence of low sperm concentration occasionally impeded ultrastructural assessment in some participants, thereby illustrating the common phenotypic overlap among quantitative, motility, and morphological sperm defects [43]. More recent studies have similarly identified variants affecting dynein- and axoneme-associated proteins in men diagnosed with oligoasthenoteratozoospermia. Biallelic *DNAH3* variants were associated with an OAT phenotype and defective sperm flagellar structure [44], while a *CCDC34* variant was linked to impaired axonemal organization and altered outer dynein-arm assembly or stability [45]. Variants in *TTC12*, another cilia-associated gene, have also been reported in a man with severe OAT and primary ciliary dyskinesia [46].

Importantly, although many ciliary and axonemal genes have traditionally been associated with sperm motility disorders, a growing body of evidence suggests that these proteins are also integral to several earlier stages of spermatogenesis. Cytoskeletal and dynein-associated proteins can also participate in germ-cell development, intracellular transport, spermiogenesis, spermiation, and maintenance of the seminiferous epithelium [41,47]. Consequently, disruption of these pathways may impair not only sperm motility but also sperm production, thereby providing a plausible biological explanation for their enrichment in oligozoospermic individuals. This broader role is exemplified by *AXDND1*: damaging variants were identified through next-generation sequencing in males presenting with diminished or absent sperm counts, while functional studies in knockout mice revealed deficiencies in spermatogonial maintenance, germ-cell survival, spermiation, sperm individualization, and axonemal structure. Thus, disruption of a dynein-related protein affected both sperm production and flagellar function [48]. Similarly, loss of *CEP70*, a centrosomal and cilia-associated gene, impeded acrosome and flagellum formation, augmented germ-cell apoptosis, and produced an OAT phenotype in experimental models, thereby further illustrating how cytoskeletal disruption can concurrently affect sperm count, morphology, and motility [49]. Studies focused on severe oligozoospermia and azoospermia also corroborate the broader concept that reduced sperm concentration may arise from defects affecting multiple stages of spermatogenesis, rather than from a single molecular pathway. Clinical exome analysis has identified putative genetic etiologies in men with severe oligozoospermia, including truncating variants in *BNC1* [50], whereas recent functional studies have demonstrated that biallelic *YTHDC2* variants may cause either non-obstructive azoospermia or severe oligozoospermia by disrupting the mitotic-to-meiotic transition [51]. These findings underscore the substantial genetic heterogeneity of quantitative spermatogenic failure.

In this context, the present results extend previous gene-centered studies by identifying pathway-level convergence on the microtubule–axoneme–cilium axis in Greek men with oligozoospermia. Although functional validation is required, the agreement among GO, KEGG, and STRING analyses supports the hypothesis that alterations affecting cytoskeletal architecture and ciliary machinery may contribute not only to abnormal sperm movement or morphology but also to impaired sperm production.

It should be noted however, that the convergence of GO, KEGG, and STRING analyses on cytoskeletal, microtubule, axonemal, and ciliary processes represents an interesting biological pattern within the prioritized gene set. These analyses were performed downstream of the same candidate-prioritization framework and are therefore not independent lines of evidence for a disease mechanism. Rather, their convergence highlights biological themes that may warrant further investigation and should be considered hypothesis-generating until supported by independent genetic and functional evidence.

### 4.2. Biological Relevance of Prioritized Candidate Genes

To facilitate interpretation of the prioritized genes according to the strength of the currently available evidence, they are discussed separately below as (i) genes with previous evidence of involvement in male infertility, spermatogenesis, or related reproductive phenotypes and (ii) less-characterized candidate genes emerging from the present analysis.

#### 4.2.1. Genes Previously Implicated in Male Infertility and Spermatogenesis

Genes with previous evidence of involvement in male infertility were identified among the prioritized candidates. Among the prioritized genes, the axonemal dynein heavy-chain genes *DNAH5*, *DNAH11*, and *DNAH12* were particularly notable, as they collectively accounted for the significant enrichment of minus-end-directed microtubule motor activity and contributed to several cilium- and axoneme-related GO categories. Dynein heavy chains are force-generating components of axonemal motor complexes, although their distribution and function may differ between respiratory cilia, reproductive-tract cilia, and sperm flagella [52,53]. This distinction is especially relevant for *DNAH5*. Loss-of-function variants in this gene are a well-established cause of primary ciliary dyskinesia with outer dynein-arm defects [54]. However, translational studies in humans and mice have also shown that *DNAH5* deficiency can cause oligozoospermia through impaired motility of efferent-duct cilia, resulting in defective sperm transport despite largely preserved sperm flagellar ultrastructure and motility [55]. This finding provides a particularly compelling biological link between *DNAH5* and reduced sperm concentration, rather than solely asthenozoospermia.

Recent evidence also supports direct roles for *DNAH11* and *DNAH12* in human male infertility. Biallelic *DNAH11* variants have been associated with abnormal sperm flagellar morphology, defective dynein-arm organization, asthenozoospermia, and male infertility [56,57], while earlier population-based evidence linked *DNAH11* polymorphisms with increased susceptibility to asthenozoospermia [58]. Similarly, independent human and mouse studies have demonstrated that *DNAH12* is required for normal sperm flagellar development. Its deficiency disrupts inner dynein-arm assembly and axonemal organization in sperm, leading to severe motility and morphological abnormalities and male infertility [59,60]. Although these genes have most often been studied in relation to qualitative sperm defects, their identification in the present oligozoospermic cohort is biologically plausible because dynein-dependent processes also influence sperm transport and may contribute to overlapping semen phenotypes.

The identification of *DNAH5*, *DNAH11,* and *DNAH12* should nevertheless be interpreted in relation to the specific phenotype investigated here. The oligozoospermic participants did not exhibit concomitant asthenozoospermia or teratozoospermia, with progressive motility, total motility, and normal morphology values remaining above the corresponding WHO lower fifth centiles. Although axonemal dynein genes are classically associated with ciliary and sperm-flagellar dysfunction, their reproductive effects may not be restricted to sperm motility. In particular, loss-of-function variants in *DNAH5* have been reported in men with reduced sperm counts and preserved sperm motility, while *Dnah5*-deficient mice similarly exhibit impaired sperm transport resulting from dysmotility of efferent-duct cilia without defects in sperm flagellar structure or motility [55]. This provides a potential mechanism linking ciliary dysfunction to reduced sperm numbers independently of primary sperm-motility defects. In contrast, evidence for *DNAH11* and *DNAH12* remains predominantly associated with motility- and flagellar-related phenotypes. Therefore, while the enrichment of axonemal and ciliary genes is biologically relevant, their contribution to isolated oligozoospermia, particularly for *DNAH11* and *DNAH12*, remains to be established and requires further investigation.

#### 4.2.2. Less-Characterized Candidate Genes Emerging from the Present Study

In addition to genes with established or previously reported links to male infertility, the present analysis highlighted less-characterized candidate genes that may warrant further investigation. *COL6A6* and *LAPTM4B* each harbored multiple prioritized variants with predicted functional relevance. Although current evidence directly linking these genes to oligozoospermia is limited, their emergence from the present prioritization framework identifies them as candidates for future validation rather than establishing their involvement in the phenotype. In *COL6A6*, the identification of both a splice-disrupting and a missense variant, together with its contribution to the enriched KEGG cytoskeletal pathway, supports its prioritization for further investigation. *COL6A6* encodes a collagen VI extracellular-matrix component. Although direct evidence linking *COL6A6* specifically to human oligozoospermia remains limited, extracellular-matrix organization is essential for Sertoli-cell differentiation, seminiferous-tubule integrity, blood–testis barrier dynamics, and germ-cell development [61]. Therefore, alterations in an extracellular-matrix structural gene could plausibly affect the testicular microenvironment required for normal spermatogenesis. Nevertheless, no direct evidence currently establishes *COL6A6* as a male-infertility gene, and its identification in the present study should be regarded as hypothesis-generating and requiring independent genetic and functional validation.

*LAPTM4B* similarly emerged as a less-characterized candidate gene of potential reproductive relevance. The gene encodes a lysosome-associated transmembrane protein implicated in critical cellular processes such as lysosomal trafficking, autophagy, membrane dynamics, and mTORC1-related signalling, all of which are essential for maintaining cellular homeostasis and facilitating protein turnover [62]. Supporting its biological relevance to the male reproductive system, *LAPTM4B* is expressed in human testicular tissue, with protein expression reported in cells of the seminiferous ducts and Leydig cells [63]. In cattle, *LAPTM4B* expression has also been demonstrated in reproductive tissues, with strong mRNA expression in the testis and immunolocalization in Sertoli cells, as well as expression in the epididymis and seminal gland [64]. Previous studies have also identified *LAPTM4B* as a gene involved in early embryonic development, where its expression was significantly reduced following inhibition of sperm-borne miR-34c, suggesting a potential role in embryo developmental competence and implantation [65]. However, these observations provide biological context rather than evidence of an association with male infertility. Direct evidence linking germline *LAPTM4B* variants to male infertility is currently lacking. Therefore, the identification of both a frameshift and a missense variant in the present study, together with the available reproductive and testicular-expression evidence, supports its prioritization as a candidate for further investigation but does not establish its involvement in oligozoospermia.

Overall, the candidate-gene findings underscore two complementary strengths of the present analysis. First, the recovery of genes with established roles in ciliary biology and male reproductive dysfunction provides biological credibility to the observed pathway enrichment. Second, the identification of less-characterized genes harboring multiple prioritized variants with predicted functional relevance generates novel hypotheses regarding extracellular-matrix organisation and lysosomal–autophagic regulation in impaired sperm production. These genes should not yet be considered causative; rather, they represent prioritized targets for validation in independent cohorts and experimental models.

### 4.3. Study Strengths, Limitations, and Future Directions

The present study has several notable strengths. To our knowledge, this is among the first whole-genome sequencing studies investigating the genetic architecture of oligozoospermia in a Greek population. This significantly expands the currently limited genomic data available from Southeastern Europe and the Balkans. Through the application of a comprehensive variant prioritization strategy, we identified both previously reported infertility-related genes and variants not previously observed in the databases examined, highlighting the ability of whole-genome sequencing to uncover rare and population-specific genetic variation that may remain undetected by targeted gene panels or SNP-based approaches. The stringent selection of idiopathic cases, comprehensive clinical and genetic exclusion criteria, repeated semen analyses, and the integration of multiple bioinformatic analyses, including functional enrichment and protein–protein interaction network analyses, further strengthened the biological relevance of the prioritized candidate genes and pathways.

Nevertheless, several limitations should be acknowledged. The relatively small sample size constrains the statistical power of the study, while the use of pooled sequencing prevented individual genotype confirmation and segregation analyses. Participant recruitment was based on voluntary enrollment and stringent phenotypic selection, with the aim of including individuals presenting with isolated oligozoospermia rather than mixed semen phenotypes, alongside the application of comprehensive clinical and genetic exclusion criteria. While this approach enabled the investigation of a more clearly defined phenotype, it inevitably restricted the number of eligible participants. Consequently, variants detected within the oligozoospermic pool cannot be assigned to specific participants and may have been present in only one or a subset of the five individuals comprising the pool. Furthermore, although the variants not previously observed in the databases examined were prioritized based on predicted functional impact, evolutionary conservation, and allele frequency, their absence from these resources does not establish either true novelty or pathogenicity. Such variants may represent rare or population-specific genetic variation, particularly in underrepresented populations for which genomic reference data remain limited. Consequently, these variants should be considered candidates for further investigation until validated in independent cohorts and supported by functional studies. Importantly, variants detected only in the oligozoospermic group, and not in the normozoospermic group, should be interpreted as group-specific within the present dataset rather than statistically associated with oligozoospermia. Given the relatively small cohort and pooled sequencing design, their absence from the normozoospermic dataset alone does not establish an association with the phenotype. In addition, differences in allele representation and the inherent sensitivity of pooled variant detection, particularly for low-frequency alleles, may have contributed to some of the observed group-specific differences despite the application of minimum coverage and read-support criteria. Moreover, the pooled design precluded individual-level genotype information and therefore did not permit reliable estimation of study-specific allele frequencies, effect sizes, or conventional genotype-based case-control association analyses. Instead, the group-specific comparison served as an initial filtering step, followed by prioritization based on functional impact, population frequency, and in silico analysis to identify candidate variants of potential biological relevance. Targeted validation in the original individual DNA samples would be required to determine the carrier distribution of the prioritized variants, followed by validation in larger, individually sequenced cohorts to determine whether these variants are enriched in oligozoospermic men and to formally assess their association with the phenotype. Furthermore, potential confounding factors were considered through the application of strict clinical exclusion criteria and the evaluation of demographic and lifestyle characteristics, including age, BMI, smoking status, and alcohol consumption, for which no significant differences were observed between the study groups. However, given the limited cohort size, the statistical comparisons of these characteristics cannot establish equivalence or adequate matching between the study groups, and numerical differences should be considered when interpreting the findings. Moreover, given the multifactorial nature of oligozoospermia and the limited cohort size, the potential influence of residual confounding cannot be excluded. In particular, unmeasured or incompletely characterized factors, such as hormonal status, may have contributed to the observed phenotypic and genetic differences between the groups. An additional limitation relates to the assessment of population structure. Because WGS was performed on pooled genomic DNA rather than on individually sequenced samples, individual-level genotype data were not available for formal genetic ancestry analysis, including principal component analysis. All participants were of self-reported Greek ancestry, and information on place of birth and geographic origin was collected through the study questionnaire. Previous genome-wide characterization of Greek populations has indicated relative genetic homogeneity within the general Greek population, which is positioned in proximity to other European populations, while specific geographically or culturally isolated Greek populations may exhibit distinct patterns of genetic drift [66]. Nevertheless, self-reported ancestry and geographic-origin information, together with population-level evidence, cannot substitute for individual-level genetic ancestry assessment. Therefore, residual population substructure cannot be completely excluded and may have contributed to some of the group-specific genetic differences observed. Future studies using larger cohorts and individually sequenced samples should incorporate formal ancestry analysis and appropriately population-matched controls to further address this potential source of variation. Furthermore, the present WGS analysis focused on nuclear SNPs and indels and did not include dedicated analyses of noncoding variants, mitochondrial variants, structural variants, copy-number variants, or repeat expansions. These variant classes require dedicated analytical approaches and variant-specific interpretation frameworks and may represent additional sources of genetic variation relevant to male infertility. Their exclusion therefore represents a limitation of the present study, and future studies using individually sequenced samples and complementary variant-calling approaches could provide a more comprehensive characterization of the genetic architecture of oligozoospermia. It should also be noted that peripheral blood, rather than spermatozoa, was used as the source of genomic DNA, as blood-derived DNA represents a well-established source of high-quality genomic DNA for germline WGS. This selection was made within the broader research perspective of identifying candidate genetic variants in readily accessible biological material that, following appropriate validation, could potentially contribute to the development of blood-based biomarkers. Nevertheless, sperm-derived DNA could provide complementary information, particularly regarding male germline-specific alterations or tissue-specific mosaicism that may not be detectable in peripheral blood. Future studies incorporating matched blood- and sperm-derived DNA could therefore provide further insights into the genetic complexity of oligozoospermia.

Future studies involving larger cohorts from Greece and other Balkan populations, alongside transcriptomic, functional, and family-based analyses, will be essential to validate the identified candidate genes, clarify their biological role in spermatogenesis, and determine their potential clinical utility. Expanding genomic studies in currently underrepresented populations will also enhance variant interpretation and contribute to a more comprehensive understanding of the genetic architecture of male infertility across diverse ethnic backgrounds.

## 5. Conclusions

In conclusion, this exploratory pooled-WGS study identified and prioritized candidate genetic variants and genes of potential relevance to oligozoospermia in a small cohort of Greek men. WGS-based studies of male infertility in the Greek population remain limited. Previous investigations have applied WGS to specific semen phenotypes, including teratozoospermia [67] and asthenozoospermia [68]. The present study extends this population-specific genomic research to oligozoospermia, providing an initial characterization of candidate genetic variation and biological pathways of potential relevance to this phenotype in Greek men.

The applied prioritization framework identified genes previously implicated in male infertility as well as 22 coding variants not previously observed in the databases examined. Functional enrichment analyses highlighted biological processes related to cytoskeletal organization, microtubule dynamics, axonemal assembly, ciliary function, and cellular homeostasis, generating pathway-level hypotheses that may inform future investigations of oligozoospermia.

However, given the small cohort, pooled sequencing design, absence of individual-level genotype validation, and lack of formal association testing, the identified variants and less-characterized genes should be regarded as hypothesis-generating candidates rather than as variants or genes associated with oligozoospermia. Larger studies using individually sequenced, appropriately matched cohorts will be required to assess their frequency and potential association with the phenotype, while functional studies will be necessary to investigate their biological effects. Thus, the present findings provide an exploratory basis for further investigation of candidate genetic variation and biological pathways potentially relevant to oligozoospermia in Greek men.

## Figures and Tables

**Figure 1:**
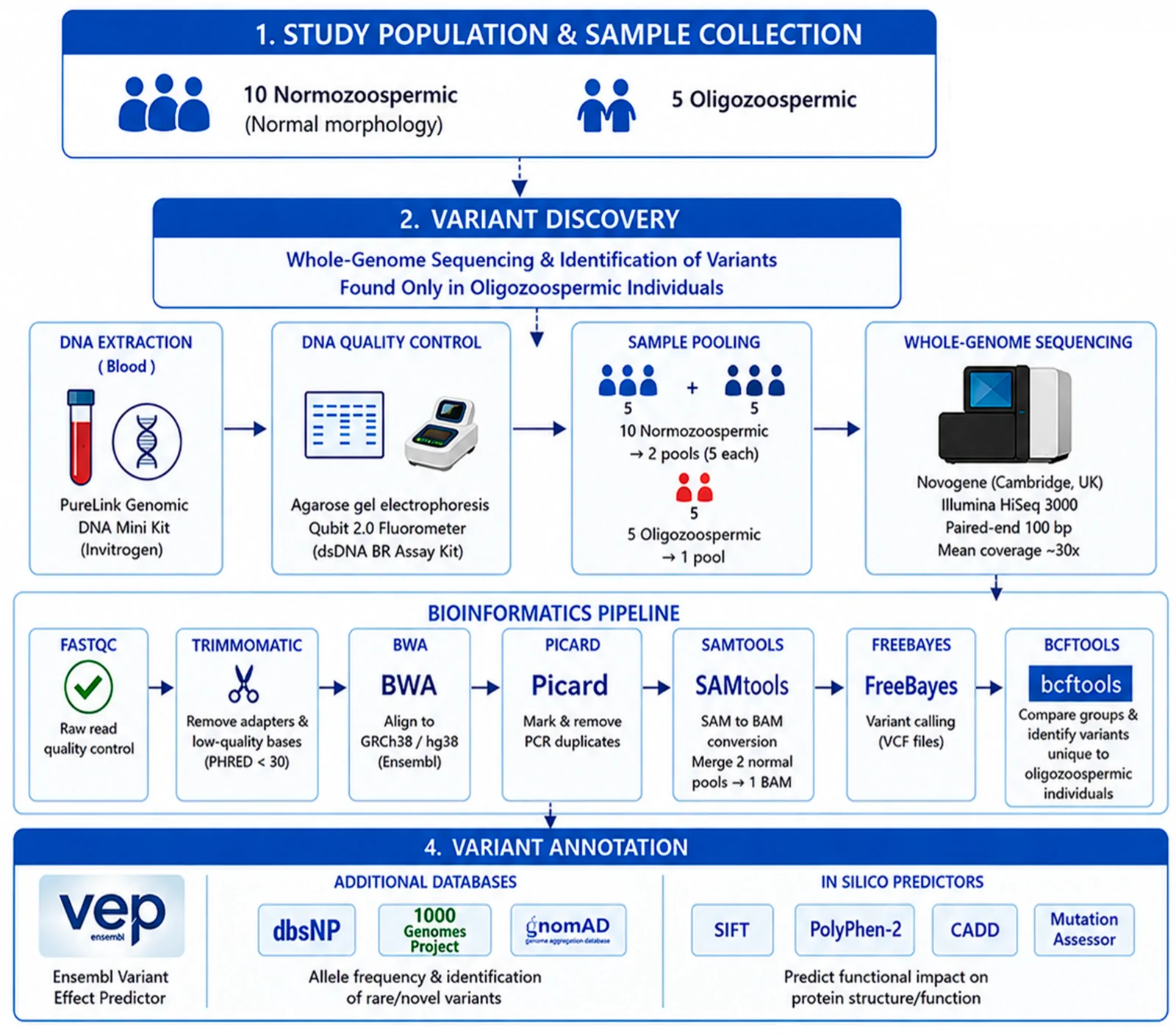
Analytical workflow for the identification and annotation of oligozoospermia-associated genetic variants in a Greek cohort.

**Figure 2:**
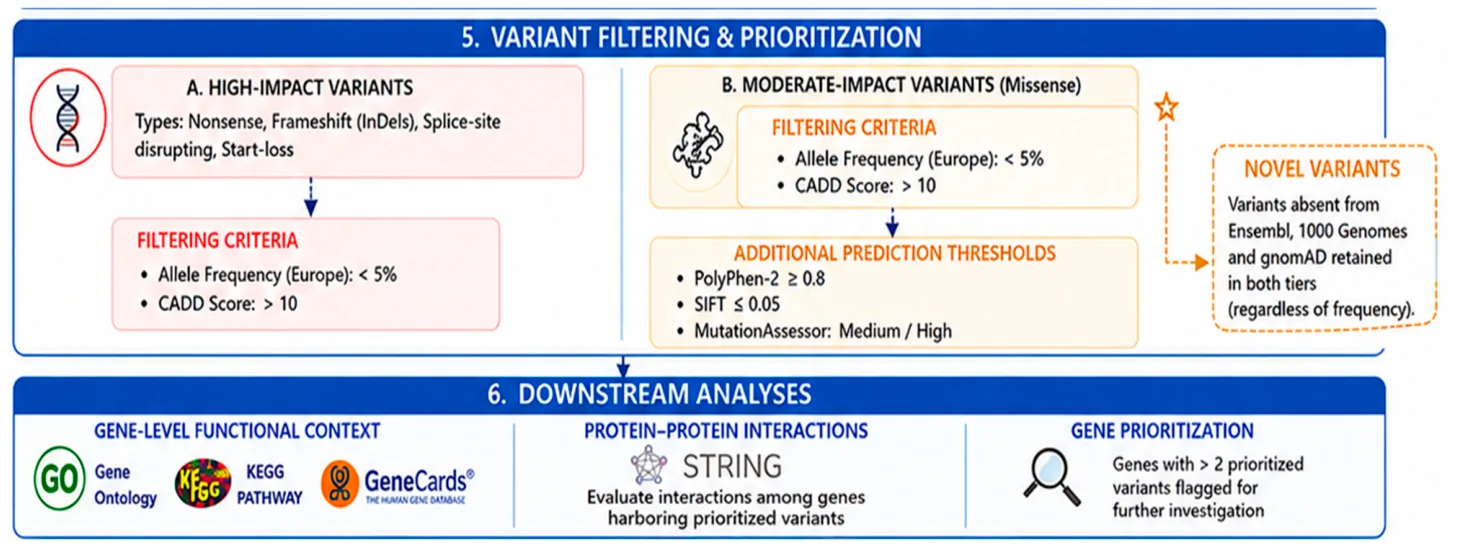
Workflow illustrating the prioritization strategy used to identify biologically relevant variants. High-impact and missense variants were filtered based on predicted functional consequences, allele frequency, CADD score, and additional in silico functional-effect prediction tools. The resulting candidate genes were functionally characterized using Gene Ontology (GO) (https://geneontology.org/), Kyoto Encyclopedia of Genes and Genomes (KEGG) (https://www.genome.jp/kegg/), GeneCards (https://www.genecards.org), and STRING (https://string-db.org/), and genes containing multiple prioritized variants were selected for downstream interpretation.

**Figure 3:**
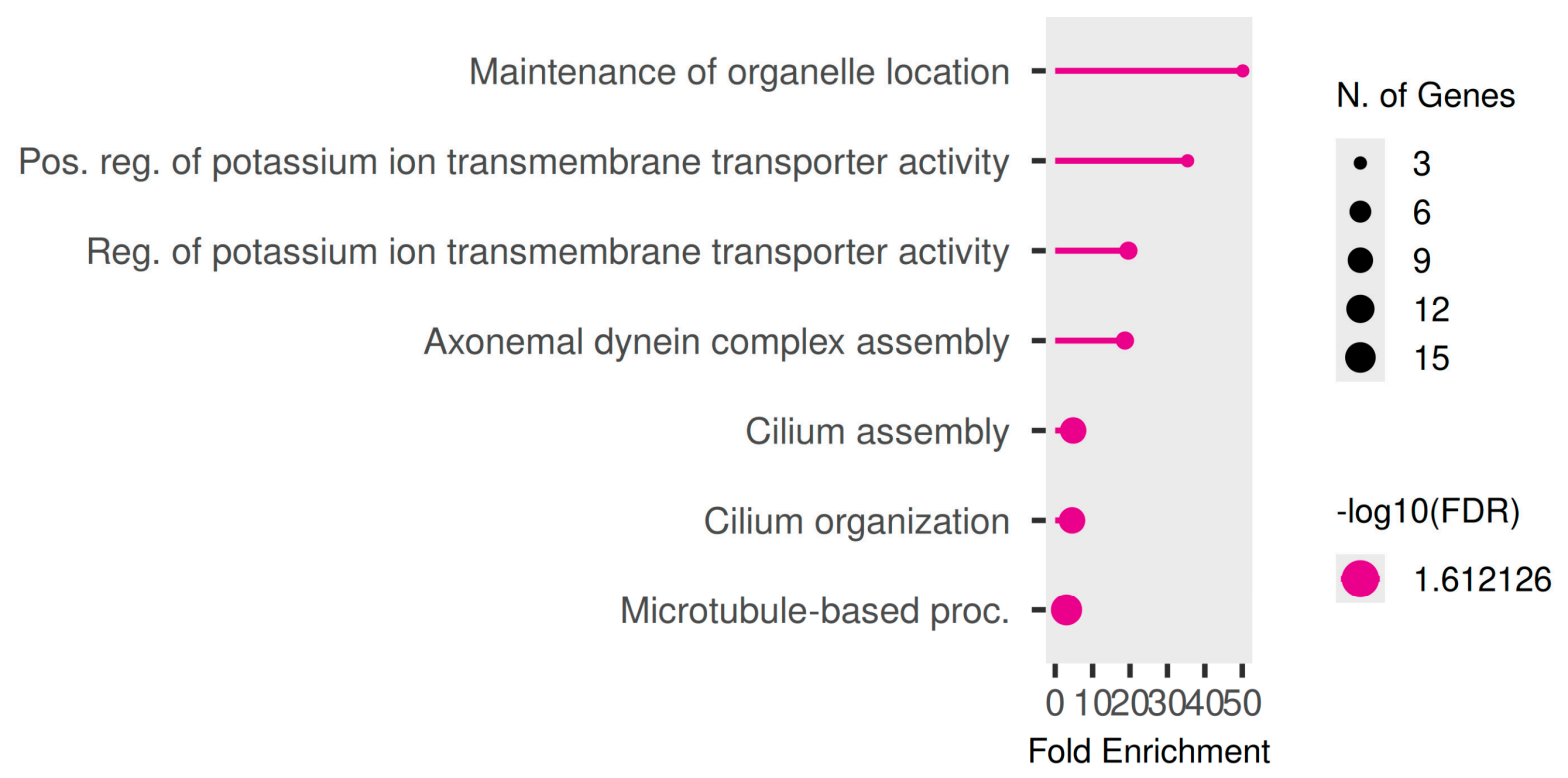
Gene Ontology (GO) Biological Process enrichment analysis of genes harboring prioritized variants. The size and color of the dots represent the number of genes and the range of the term’s statistical significance, respectively. The *y*-axis represents the GO BP terms, and the *x*-axis represents the fold enrichment. The *p*-values were corrected for multiple tests using the false discovery rate (FDR).

**Figure 4:**
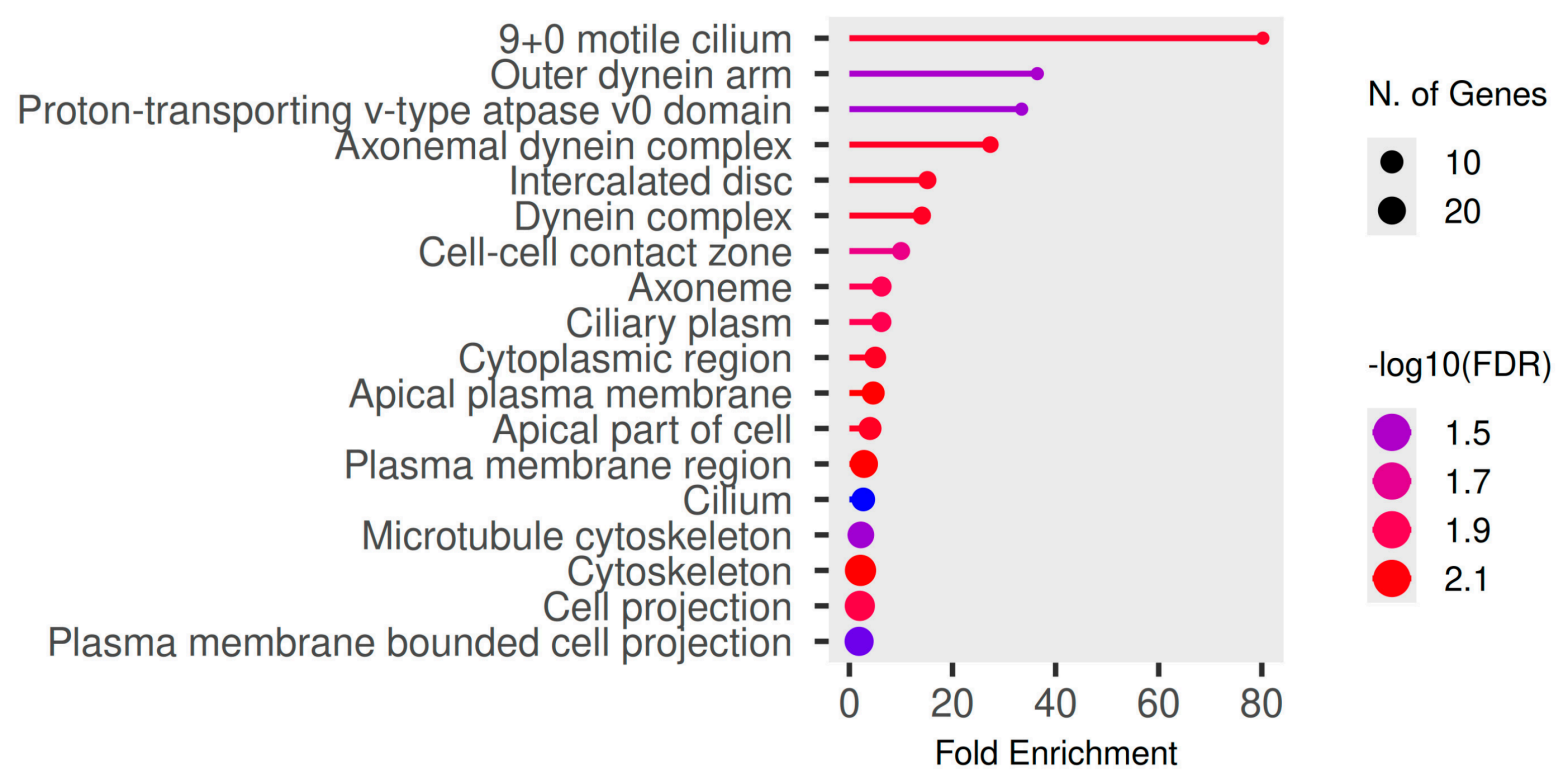
Gene Ontology (GO) Cellular Component enrichment analysis of genes harboring prioritized variants. The size and color of the dots represent the number of genes and the range of the term’s statistical significance, respectively. The *y*-axis represents the GO CC terms, and the *x*-axis represents the fold enrichment. The *p*-values were corrected for multiple tests using the false discovery rate (FDR).

**Figure 5:**
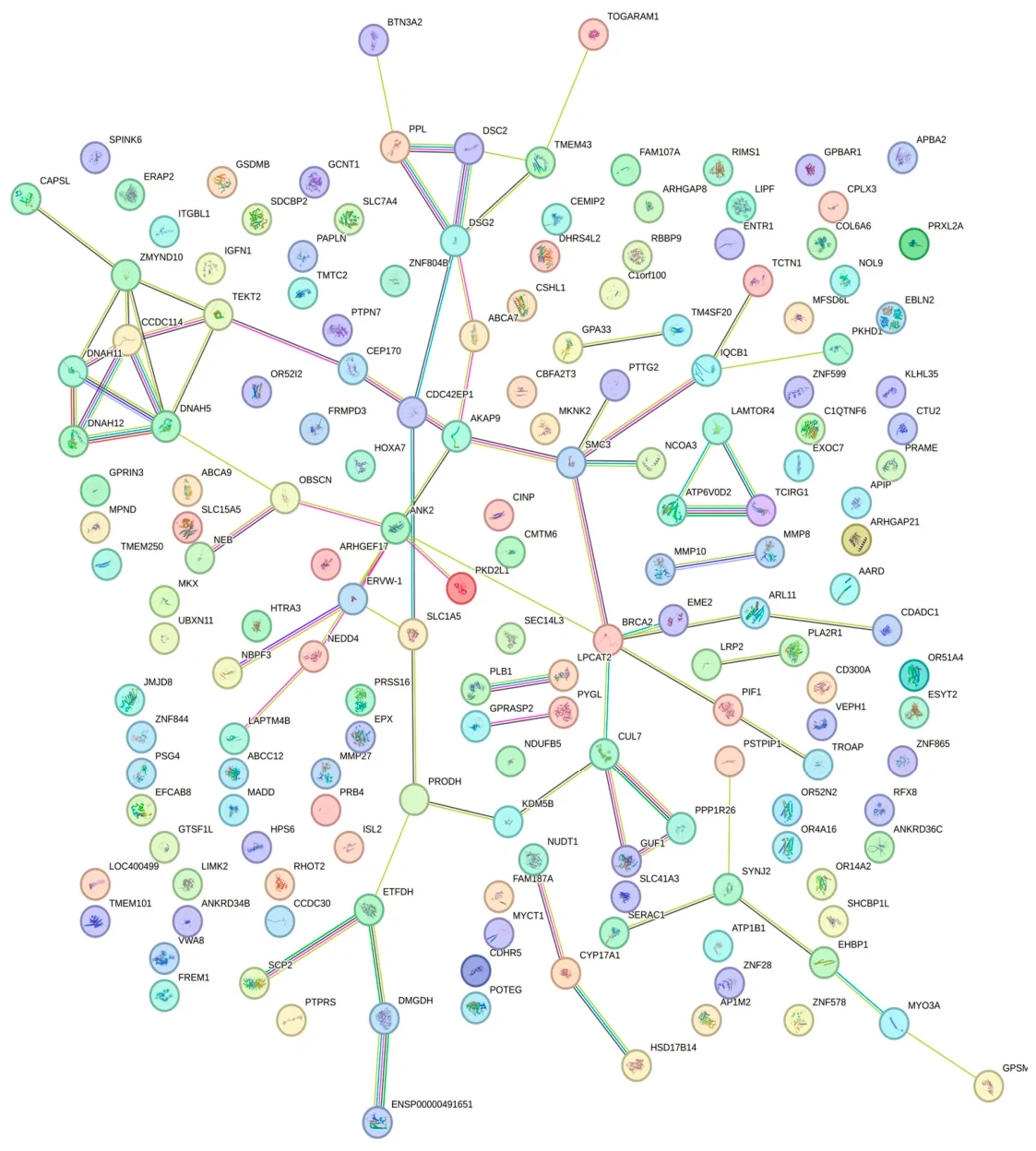
Protein–protein interaction (PPI) network generated using the STRING database for genes harboring prioritized variants identified in the oligozoospermic group. Nodes represent proteins encoded by the prioritized genes, whereas edges indicate known or predicted functional protein–protein associations rather than necessarily direct physical interactions. Edge colors denote the source of supporting evidence, including curated databases (light blue), experimentally determined interactions (magenta), gene neighborhood (green), gene fusions (red), gene co-occurrence (dark blue), text mining (yellow), co-expression (black), and protein homology (purple). Filled nodes indicate proteins with known or predicted three-dimensional structures.

**Table 1:** Demographic information of volunteers selected to participate in this study.

Demographics	Normozoospermic (*n* = 10)	Oligozoospermic (*n* = 5)	*p*-Value
Age	28–53 Mean = 36.4 (SD = 7.2)	34–41 Mean = 39 (SD = 2.83)	0.34
Body Mass Index (BMI)	19.5–40.4 Mean = 26.97 (SD = 6.07)	26.5–36.3 Mean = 31.1 (SD = 4.43)	0.16
Smoking	30% Not Smoking, 70% Smoking	40% Not Smoking, 60% Smoking	1.00
Alcohol	100% ≤ 2 drinks/week	80% ≤ 2 drinks/week	0.33
Drug Use	100% No	100% No	-
Nationality	Greek	Greek	-

**Table 2:** Significantly enriched Gene Ontology (GO) Molecular Function term identified for genes harboring prioritized variants. The table presents the enriched GO term, associated genes, fold enrichment, and false discovery rate (FDR).

GO Molecular Function	Genes	Fold Enrichment	FDR
Minus-end-directed microtubule motor activity	*DNAH5*, *DNAH11*, *DNAH12*	33.42	0.0443

**Table 3:** Significantly enriched Kyoto Encyclopedia of Genes and Genomes (KEGG) pathway identified for genes harboring prioritized variants. The table presents the enriched pathway, associated genes, fold enrichment, and false discovery rate (FDR).

KEGG Pathway	Genes	Fold Enrichment	FDR
Cytoskeleton in muscle cells (hsa04820)	*COL6A6*, *DSG2*, *ANK2*, *NEB*, *ATP1B1*, *TMEM43*, *OBSCN*	6.05	0.0178

**Table 4:** Genes harboring multiple prioritized variants identified exclusively in the oligozoospermic group.

Variant	Location	Gene	Reference	Type of Variant
-	8:98,788,168-98,788,183	*LAPTM4B*	GGGCTCCAGGCGAGGAGGGCTCCAGGCGAGG	Frameshift
rs1304930066	8:98,863,635-98,863,635	*LAPTM4B*	A	Missense
-	3:130,381,153-130,381,154	*COL6A6*	TTT	Splice Disrupting
rs59021909	3:130,285,929-130,285,929	*COL6A6*	T	Missense

## Data Availability

Whole-genome sequencing data of normozoospermic men presented in this study are available through SRA (BioProject ID PRJNA875412, http://www.ncbi.nlm.nih.gov/bioproject/875412, accessed on 3 August 2026).

## Supplementary Materials

The following supporting information can be downloaded at https://www.mdpi.com/article/10.3390/genes17101187/s1, Table S1: Individual demographic, lifestyle, and semen-analysis characteristics of the normozoospermic and oligozoospermic study participants; Table S2: Available pool-specific sequencing quality metrics; Table S3: High-impact variants identified exclusively in the oligozoospermic group; Table S4: Prioritized high-impact variants identified exclusively in the oligozoospermic group after application of the variant filtering criteria. The table includes genomic annotation, predicted functional consequence, population allele frequencies, and CADD scores; Table S5: Moderate-impact variants identified exclusively in the oligozoospermic group; Table S6: Prioritized moderate-impact variants identified exclusively in the oligozoospermic group after application of the variant filtering criteria. The table includes genomic annotation, predicted functional consequence, SIFT, Polyphen-2, MutationAssessor scores, population allele frequencies, and CADD scores.

## Abbreviations

The following abbreviations are used in this manuscript:

BP	Biological Process
CADD	Combined Annotation Dependent Depletion
CC	Cellular Component
GO	Gene Ontology
GWAS	Genome-wide Association Study
KEGG	Kyoto Encyclopedia of Genes and Genomes
MAF	Minor Allele Frequency
MF	Molecular Function
MMAF	Multiple Morphological Abnormalities of the Sperm Flagella
VEP	Variant Effect Predictor
WGS	Whole-Genome Sequencing
WHO	World Health Organization
